# Drug‐eluting stent and drug‐coated balloon for the treatment of de novo diffuse coronary artery disease lesions: A retrospective case series study

**DOI:** 10.1002/clc.24140

**Published:** 2023-09-04

**Authors:** Haobo Xu, Shubin Qiao, Jingang Cui, Jiansong Yuan, Weixian Yang, Rong Liu, Tianjie Wang, Hao Guan, Tao Tian, Fasheng Zhu, Juan Wang, Yue Chang, Zhuoxuan Yang, Shengwen Liu

**Affiliations:** ^1^ Department of Cardiology Fuwai Hospital, National Center for Cardiovascular Diseases, Chinese Academy of Medical Sciences Beijing People's Republic of China; ^2^ Department of Cardiology Yuncheng Central Hospital Shanxi People's Republic of China

**Keywords:** de novo diffuse coronary artery disease, drug‐coated balloon, drug‐eluting stent, hybrid strategy, quantitative flow ratio

## Abstract

**Background:**

The hybrid strategy of a combination of drug‐eluting stent (DES) and drug‐coated balloon (DCB) is promising for the treatment of de novo diffuse coronary artery disease (CAD).

**Hypothesis:**

To investigate the efficacy and functional results of hybrid strategy.

**Methods:**

This case series study included patients treated with a hybrid approach for de novo diffuse CAD between February 2017 and November 2021. Postprocedural quantitative flow ratio (QFR) was used to evaluate the functional results. The primary endpoint was procedural success rate. The secondary endpoints were major adverse cardiovascular events (MACE) including cardiac death, myocardial infarction (MI) (including peri‐procedural MI), and target vessel revascularization.

**Results:**

A total of 109 patients with 114 lesions were treated. DES and DCB were commonly used in larger proximal segments and smaller distal segments, respectively. The mean QFR value was 0.9 ± 0.1 and 105 patients (96.3%) had values >0.8 in all the treated vessels. Procedural success was achieved in 106 (97.2%) patients. No cases of cardiac death were reported at a median follow‐up of 19 months. Spontaneous MI occurred in three (2.8%) patients and target vessel revascularization in six (5.5%) patients. Estimated 2‐year rate of MACE excluding peri‐procedural MI was higher in the group with lower QFR value (12.1 ± 5.7% vs. 5.6 ± 4.4%, log‐rank *p* = .035) (cut‐off value 0.9).

**Conclusion:**

Hybrid strategy is a promising approach for the treatment of de novo diffuse CAD. Postprocedural QFR has some implications for prognosis and may be helpful in guiding this approach.

## Graphical abstract

A hybrid strategy using DES and DCB showed encouraging performance and acceptable clinical outcomes for the treatment of de novo diffuse CAD. This approach also yielded promising functional results as evaluated by QFR. Higher post‐PCI QFR value was associated with better prognosis and might be helpful in guidance of this approach.

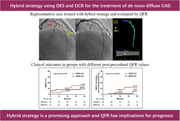

## 1 INTRODUCTION

Despite the long‐term safety and efficacy of new‐generation drug‐eluting stents (DES), concerns remain regarding the late adverse events associated with the presence of a permanent rigid metallic cage.^1^ Drug‐coated balloon (DCB) is a promising tool for the treatment of in stent restenosis (ISR) and de novo small coronary disease.^2^, ^3^ Moreover, DCB has been explored as an adjuvant tool for DES in complex lesions.^4^

Percutaneous coronary intervention (PCI) for de novo diffuse coronary artery disease (CAD) is challenging in clinical practice. Stent length is known to independently predict ISR and stent thrombosis.^5^, ^6^ Furthermore, stenting with long overlapping DES is associated with a high adverse events rate.^7^, ^8^ And, the implantation of long metal devices limits access for coronary artery bypass graft. A hybrid procedure combining DES implantation (located in the larger, more proximal part of the lesion) and DCB inflation (located in the smaller distal segment) has proven to be an alternative and useful approach for treating de novo diffuse CAD in studies with small sample size.^9^, ^10^ However, additional clinical data are needed to confirm the efficacy of this approach. Furthermore, no information is available on whether promising angiographic results reflect a similar effectiveness in functional results.

The quantitative flow ratio (QFR), derived from three‐dimensional coronary artery reconstruction and fluid dynamics computations from the angiogram, enables online estimation of the fractional flow reserve without the use of a pressure wire or adenosine‐induced maximal hyperemia.^11^ In the present study, we evaluated the preliminary results of a hybrid strategy for treating de novo coronary diffuse lesions in a relatively large cohort and used QFR to investigate whether this strategy yielded promising functional results.

## 2 MATERIALS AND METHODS

### 2.1 Implementation of hybrid strategy

In our study, a hybrid strategy was defined as the use of slightly overlapping new‐generation DES implantation (usually located in the larger, more proximal part of the lesion) and DCB inflation (usually located in the smaller distal segment of the same lesion) for de novo diffuse CAD lesions. Spot DES implantation for the requisite part of the DCB‐treated segment, but that does not completely cover the segment was also considered as a hybrid strategy. Lesions that were initially treated with DCB and subsequently underwent bailout stenting, which completely covered the DCB‐treated segment, were not considered as being treated with the hybrid strategy. The decision to perform “hybrid strategy” rather than “conventional” permanent metallic stent implantation was left to the operator's discretion in the presence of the aforementioned lesion characteristics. In the DCB‐treated segment, bail‐out stenting was considered with signs of coronary dissections greater than or equal to type C (National Heart, Lung, and Blood Institute [NHBLI] classification system for intimal tears, developed by the Coronary Angioplasty Registry) or reduced flow (Thrombolysis In Myocardial Infarction [TIMI] flow grade less than 3), or when the residual stenosis was evaluated to be >30%. The interventional approach, intravascular ultrasound or optical coherence tomography, and administration of glycoprotein IIb/IIIa receptor inhibitors during the procedure were performed at the discretion of the operator. All study patients received standard medical therapy including statins, aspirin, clopidogrel or ticagrelor, beta‐blockers, angiotensin‐converting enzyme inhibitors/angiotensin receptor blockers, calcium channel blockers, and oral nitrates after the initial admission. Patients received standard double antiplatelet therapy before the procedure, which was continued for 12 months.

### 2.2 Study population

This retrospective case series study did not include a control group. Patients who underwent PCI using the hybrid strategy for chronic or acute coronary syndrome due to de novo diffuse CAD between February 2017 and November 2021 at Fuwai Hospital were enrolled. De novo coronary diffuse lesion was defined as a lesion with a length >25 mm involving small distal segment with a reference vessel diameter (RVD) >2.0 and ≤2.75 mm. Lesions with ISR and chronic total occlusion and those with bifurcation lesions with DES implanted in the main branch and DCB inflated in the side branch were excluded. On the basis of these criteria, 109 patients with 114 lesions were included in this study. All patients were carefully informed of alternative treatment options and provided written informed consent for the procedure. The study was approved by the Ethics Committee of Fuwai Hospital and was conducted in accordance with the ethical principles stated in the Declaration of Helsinki.

### 2.3 Quantitative coronary angiography (QCA) analysis and QFR computation

QCA, including assessment of reference vessel diameter, minimal lumen diameter, diameter stenosis, lesion length, and acute gain was analyzed by a blinded independent core laboratory (CCRF) using well‐validated software (QAngio software version 7.3; Medis Medical Imaging Systems) as previously described.^3^

QFR measurement was performed postprocedural in patients with analyzable angiogram. QFR was analyzed from the ostium of the main vessels (left anterior descending, left circumflex, and right coronary artery) to a landmark distal to the farthest measurement‐requiring lesion. Briefly, two angiographic images with angles ≥25° apart were required for QFR computation. The lumen contour was automatically delineated by extensively validated algorithms. Manual correction was allowed in case of suboptimal angiographic image quality, following a standard operation procedure. The reference vessel diameter was generally obtained by selecting the automatic reference interpolation mode. The contrast flow model, which uses frame counting to derive contrast flow velocity from the angiogram, was used in this study for QFR computation. Off‐line QFR analysis was performed by well‐trained technicians from a blinded independent core laboratory (CCRF), using QFR system (AngioPlus; Pulse Medical Imaging Technology). The cutoff value of QFR for physiological significance was defined as 0.80. Two independent operators, blinded to outcomes, performed QFR computations. Both are certified operators for QFR computation. The interrater agreement between operators was very high in all cases (*k* > 0.95).

### 2.4 Definitions and clinical outcomes

Clinical follow‐up was achieved for all recruited subjects by clinic visit or telephone interview. Angiographic follow‐up was not routinely performed unless clinically indicated or as part of a separate revascularization procedure. The primary endpoint of the study was procedural success, defined as a residual stenosis less than 30% at any‐treated segment with TIMI flow grade 3 without in‐hospital cardiac death, target vessel Q‐wave myocardial infarction (MI) or need for emergent target lesion revascularization (TLR). The secondary endpoints were major adverse cardiovascular events (MACE) including cardiac death, MI (including peri‐procedural MI), and target vessel revascularization (TVR). Other outcomes included procedural success with post‐PCI QFR value >0.8 in any treated vessels, spontaneous MI, definite/probable stent thrombosis, and TLR. Clinical events were defined according to the Academic Research Consortium‐2 definitions.^12^

### 2.5 Statistical analysis

The results were expressed as mean ± standard deviation, or number (percentage). Differences of continuous variables between groups were compared using Student unpaired *t* test or Mann–Whitney *U* test, as appropriate. Comparison of categorical variables was performed using the *χ* ^2^ or Fisher exact test, as appropriate. Patients were grouped according the presence or not of a treated‐vessel with a post‐PCI QFR value <0.9. The time‐to‐event rates for groups were estimated using Kaplan–Meier methods and were compared by the log‐rank test. All reported probability values were 2‐tailed, and a *p* < .05 was considered statistically significant. SPSS version 24.0 (IBM Corp) was used for calculations and illustrations.

## 3 RESULTS

### 3.1 Baseline patient characteristics

**Figure 1:**
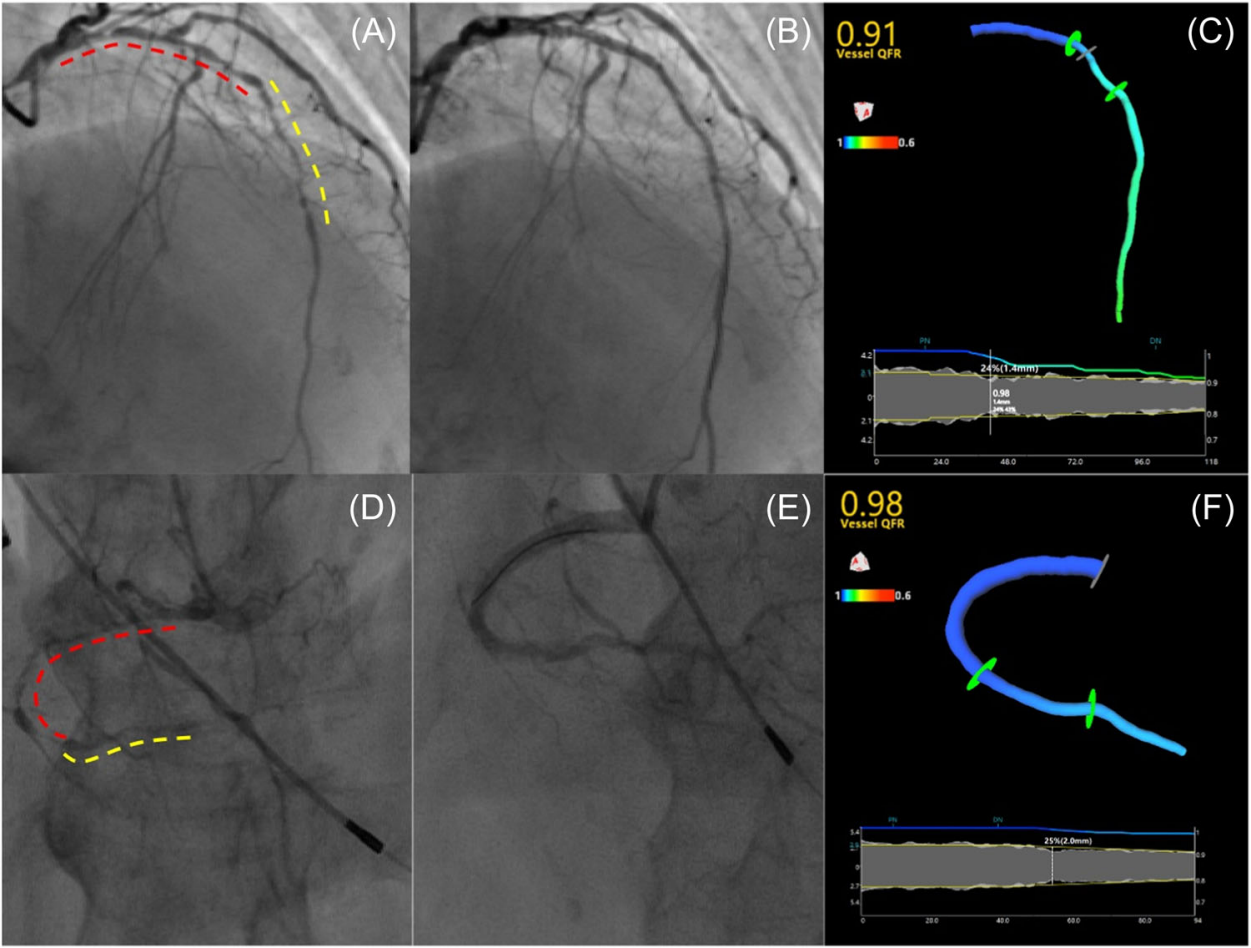
Two cases of hybrid PCI approach for de novo long diffuse coronary lesions. Case 1: (A) Angiogram in a patient with a de novo long diffuse lesions involving proximal‐mid left anterior descending. DES‐ and DCB‐treated segments were shown in red and yellow dotted line respectively. (B) Final angiographic result. (C) A 3D model of the coronary was reconstructed based on post‐PCI angiogram and result showed an optimal post‐PCI QFR value of 0.91. Case 2: (D) Angiogram in a patient with a de novo long diffuse lesions involving proximal‐distal right coronary artery. DES‐ and DCB‐treated segments were shown in red and yellow dotted line respectively. (E) Final angiographic result. (F) A three‐dimensional model of the coronary was reconstructed based on post‐PCI angiogram and result showed an optimal post‐PCI QFR value of 0.98. DCB, drug‐coated balloon; DES, drug‐eluting stent; PCI, percutaneous coronary intervention; QFR, quantitative flow ratio.

A total of 109 patients were treated with the hybrid strategy for de novo diffuse CAD. One patient underwent bail‐out stenting because of type C dissection in the DCB‐treated segment. Three patients underwent bail‐out stenting because of type C dissection after an initial long‐length DCB‐only strategy. In these four patients, spot DES was implanted in the DCB‐treated segment but without completely covering the segment. Therefore, these patients were included in the present study. Representative cases were shown in Figure 1. Baseline patient demographic characteristics were presented in Table 1. Mean age was 59.9 ± 10.2 years and 71.6% of the patients were male. Mean SYNTAX score was 21.7 ± 9.1.

**Table 1:** Baseline demographic and procedural characteristics.

Items	Patients, *n* = 109
Age (years)	59.9 ± 10.2
Male	78 (71.6)
BMI (kg/m^2^)	25.8 ± 2.8
Hypertension	78 (71.6)
Hyperlipidemia	92 (84.4)
Diabetes	58 (53.2)
Current smoker	49 (45.0)
Family history of CAD	26 (23.9)
Prior MI	24 (22.0)
Prior stroke	13 (11.9)
Prior PCI	24 (22.0)
Prior CABG	3 (2.8)
Acute coronary syndrome	40 (36.7)
Multivessel CAD	103 (94.5)
SYNTAX score	21.7 ± 9.1
LVEF (%)	60.2 ± 8.0
NT‐pro BNP (pg/mL)	316.0 ± 501.0
LDL‐C (mmol/L)	2.2 ± 0.8

Items	Lesions, *n* = 114
Target vessel	
Left anterior descending	47 (41.2)
Left circumflex	36 (31.6)
Right coronary artery	31 (27.2)
Location of lesion in treated vessel	
Proximal involved	49 (43.0)
Mid/distal	65 (57.0)
Balloon predilation	114 (100.0)
Intracoronary imaging	
IVUS/OCT	16 (14.0)
Hybrid strategy	
DES proximal with DCB distal	92 (80.7)
DCB proximal with DES distal	22 (19.3)
Device characteristics	
Number of DES per lesion	1.5 ± 0.7
Number of DCB per lesion	1.1 ± 0.3
DES length per lesion (mm)	36.4 ± 19.3
DCB length per lesion	25.3 ± 10.3
DES diameter per lesion	2.7 ± 0.4
DCB diameter per lesion	2.4 ± 0.4
TIMI flow grade	
Preprocedural TIMI flow grade 3	74 (64.9)
Postprocedural TIMI flow grade 3	114 (100.0)
Dissection postprocedural	31 (27.2)

*Note*: Values are expressed as mean ± SD or *n* (%).

Abbreviations: BMI, body mass index; CABG, coronary artery bypass graft; CAD, coronary artery disease; DCB, drug‐coated balloon; DES, drug‐eluting stent; IVU, intravascular ultrasound; LDL‐C, low‐density lipoprotein cholesterol; LVEF, left ventricular ejection fraction; MI, myocardial infarction; NT‐pro BNP, N‐terminal pro‐B‐type natriuretic peptide; OCT, optical coherence tomography; PCI, percutaneous coronary intervention; SYNTAX, synergy between percutaneous coronary intervention with taxus and cardiac surgery; TIMI, thrombolysis in myocardial infarction.

### 3.2 Angiographic and procedural details

Angiographic and procedural characteristics were summarized in Table 1. A total of 114 lesions were included. Regarding the types of DCB, SeQuent Please (B. Braun) was used for 70 lesions, Bingo (Yinyi) for 48 lesions, and Restore (Cardionovum) for two lesions. The hybrid strategy was performed as a proximal DES with distal DCB in the majority of lesions (80.7%). Proximal DCB with distal DES was performed for the remaining lesions (19.3%) (Supporting Information: Figure 1). The mean number of DES per lesion was greater than that of DCB. Postprocedural TIMI flow grade 3 was achieved in all the treated vessels. The results of QCA were summarized in Table 2. The reference vessel diameter and lesion length of the DES‐treated segments were greater than those of the DCB‐treated segments. Compared with the DCB‐treated segment, the DES‐treated segment had better postprocedural results, with less residual stenosis and more acute gain.

**Table 2:** QCA measurements in the treated segments at baseline and after the procedure.

Items	DES‐treated segment	DCB‐treated segment	*p* Value
	*n* = 114	*n* = 114	
*Preprocedural QCA*			
Reference vessel diameter (mm)	2.7 ± 0.3	2.3 ± 0.3	<.001
Minimal lumen diameter (mm)	0.4 ± 0.2	0.4 ± 0.2	.721
Diameter stenosis (%)	83.6 ± 7.4	81.5 ± 6.8	.023
Lesion length (mm)	33.0 ± 16.3	23.3 ± 8.9	<.001
*Postprocedural QCA*			
Minimal lumen diameter (mm)	2.4 ± 0.2	1.9 ± 0.3	<.001
Diameter stenosis (%)	10.7 ± 2.9	18.4 ± 7.3	<.001
Acute gain (mm)	1.9 ± 0.3	1.5 ± 0.3	<.001

*Note*: Values are expressed as means ± SD.

Abbreviations: DCB, drug‐coated balloon; DES, drug‐eluting stents; QCA, quantitative coronary angiography.

### 3.3 Post‐PCI QFR measurement

All patients were eligible for post‐PCI QFR measurements. The mean post‐PCI QFR value was 0.9 ± 0.1. The distribution of QFR values was shown in Supporting Information: Figure 2. One hundred and ten vessels (96.5%) and 105 patients (96.3%) had a QFR > 0.8, while 77 vessels (67.5%) and 73 patients (67.0%) had a QFR > 0.9. There were no significant differences in the demographic characteristics, lesions, and procedural characteristics between groups stratified based on the presence of vessels with a QFR value <0.9 (Supporting Information: Tables 1 and 2). The group with QFR value ≥0.9 had shorter lesion length and less post‐PCI percentage of diameter stenosis (%DS) in DCB‐treated segment than the group with QFR value <0.9 (Supporting Information: Table 3). Lesion length and post‐PCI %DS in the DCB‐treated segments were also significantly correlated with post‐PCI QFR values (Supporting Information: Table 4). In multivariate regression analysis, only post‐PCI %DS in the DCB‐treated segment (odds ratio 1.13, 95% confidence interval 1.06–1.21, *p* < .001) was found to be a significant risk factor for lower post‐PCI QFR values (Supporting Information: Table 5).

### 3.4 Clinical outcomes

**Figure 2:**
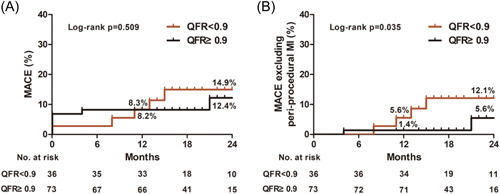
Kaplan–Meier time‐to‐event curve for MACE (A) and for MACE excluding peri‐procedural myocardial infarction (B) according to postpercutaneous coronary intervention QFR cut‐off value (0.9). MACE, major adverse cardiovascular events; MI, myocardial infarction; QFR, quantitative flow ratio.

Procedural success was achieved in 106 patients (97.2%) (Table 3). The remaining three patients were considered unsuccessful because the post‐PCI %DS in the DCB‐treated segment was >30% (Supporting Information: Figure 3). Procedural success with QFR value >0.8 in any treated vessels was achieved in 105 (96.3%) patients. Peri‐procedural MI occurred in six patients (5.5%), while in‐hospital cardiac death and stent thrombosis were not recorded. All patients had at least one follow‐up contact (median, 19.0 months; interquartile range, 16.0–24.0 months). No cases of death were reported at follow‐up. TVR occurred in six patients (5.5%) and spontaneous MI occurred in three patients (2.8%). Among the TVR cases, TLR occurred in three patients (2.8%) (one had restenosis in the DCB‐treated segment and was treated with DES, one had very late stent thrombosis and was treated with thrombus aspiration followed by plain old balloon angioplasty, and one had in‐stent restenosis and was treated with DCB), while others underwent revascularization due to the progression of nontarget lesions. Because a previous study indicated that a lower post‐PCI QFR value (<0.9) predicted subsequent adverse events, we conducted further analyses. Estimated MACE rate at 2‐year follow‐up (Figure 2A,B) was similar between groups with lower QFR value (<0.9) and higher QFR value (≥0.9) (14.9 ± 6.2% vs. 12.4 ± 5.1%, *p* = .509), but the estimated rate of MACE excluding periprocedural MI was higher in the group with lower QFR value (12.1 ± 5.7% vs. 5.6 ± 4.4%, *p* = .035).

**Table 3:** Cumulative clinical events following hybrid strategy.

	Patients, *n* = 109
Procedural success	106 (97.2)
Procedural success with QFR value more than 0.8 in all treated vessels	105 (96.3)
In‐hospital events	
Cardiac death	0 (0.0)
Periprocedural MI	6 (5.5)
ST (definite/probable)	0 (0.0)
Follow‐up events	
Cardiac death	0 (0.0)
TVR	6 (5.5)
Spontaneous MI	3 (2.8)
MACE	13 (11.9)
MACE excluding periprocedural MI	7 (6.4)
ST (definite/probable)	2 (1.8)
TLR	3 (2.8)

*Note*: Values are presented as *n* (%). MACE is defined as cardiac death, MI (including peri‐procedural MI) and TVR.

Abbreviations: MACE, major adverse cardiac events; MI, myocardial infarction; QFR, quantitative flow ratio; ST, stent thrombosis; TLR, target lesion revascularization; TVR, target vessel revascularization.

## 4 DISCUSSION

In this study, a total of 109 patients with 114 de novo diffuse CAD lesions were treated using hybrid strategy. Procedural success was achieved in 106 patients (97.2%). The mean QFR value was 0.9 ± 0.1 and 105 patients (96.3%) had a QFR value >0.8 in all the treated vessels. Our study is the first to provide strong evidence for the physiological efficacy of hybrid strategy in de novo diffuse CAD.

Currently, DCB treatment is recommended for the treatment of ISR in bare‐metal stents or DES, which deserves a Class Ia recommendation from the European Society of Cardiology.^13^ Moreover, the role of DCB has been tested in several other settings such as de novo large vessel disease, multivessel disease, and long diffuse coronary disease, with promising results regarding safety and effectiveness.^4^ However, the DCB‐only strategy may be limited in very complex lesions owing to severe coronary dissection after dilation and residual diameter stenosis. Under these circumstances, a hybrid strategy consisting of the use of DES and DCB, with the aim of reducing the amount of implanted metal and minimizing the risk of ISR and stent thrombosis, could be a solution. In a study by Costopoulos et al.^9^ involving the use of DCB in 93 de novo diffuse CAD lesions, the DCB‐only strategy was used in 52 (56.0%) lesions, the hybrid strategy in 34 (36.6%) lesions, and DEB with DES bail‐out in 7 (7.4%) lesions. The lesion length treated in hybrid approach (67.7 ± 13.4 mm) was much longer than that in DCB‐only strategy (35.4 ± 5.7 mm), which indicated that hybrid strategy has the potential to treat more complex lesions than DCB‐only strategy. The hybrid approach has also been used in other settings such as the combination of a bioresorbable scaffold and DCB for the treatment of diffuse CAD,^14^ and DES in the main branch with DCB in the side branch for the treatment of bifurcation lesion.^10^

This study included 109 patients (114 lesions) and was the largest study on the use of hybrid strategy for the treatment of de novo diffuse CAD. In our population, multivessel disease was present in most patients and mean SYNTAX score was 21.7 ± 9.1 which was in accordance with that in previous studies.^9^, ^14^ Besides, the approach was primarily performed as DES proximal with DCB distal and the lesion length was especially long (exceeding 55 mm with 33.0 ± 16.3 mm in the DES‐treated segment and 23.3 ± 8.9 mm in the DCB‐treated segment), which was in line with that observed in previous reports.^9^ Additionally, the results of our study were consistent with those of Ielasi et al.^14^ who showed a high procedural success rate. Taken together, these preliminary results showed that the hybrid approach could be an alternative for treating de novo diffuse CAD. Notably, bail‐out stenting occurred in only one patient treated with the hybrid strategy, while the rate of bail‐out stenting was nearly 7.4% in a study by Costopoulos et al.^9^ This could be explained by the relatively shorter length and smaller lumen diameter in the DCB‐treated segment in our study, as well as variations in the procedures by different operators.

With regard to the clinical outcomes of de novo diffuse lesions after PCI, a large single‐center experience previously demonstrated that treatment with >60 mm overlapping permanent DES, although associated with acceptable mortality and ST rates, can lead to high TLR rates (approximating 24%) at the 3‐year follow‐up.^7^ In the LONG DES trials, in which patients with diffuse disease (≥25 mm) were treated with only DES, the 1‐year TVR and MACE rates were 3.3% and 12.2% in LONG DES III, 2.2% and 15.2% in IV, and 2.8% and 16.6% in V, respectively.^8^, ^15^, ^16^ In our study, the TVR rate was 5.5% after a median follow‐up of 19 months, which was higher than that reported in the LONG DES trials. It is important to note that in the LONG DES trials, vessels with larger diameter (mean reference vessel diameter 3.2 ± 0.4 mm) and shorter length (nearly 30 mm) were treated and the prevalence of diabetes mellitus was lower (about 30% of the population). In addition, the TVR rates in the LONG DES trials were from the 1‐year follow‐up period, which was shorter than the follow‐up duration in our study. Interestingly, the TVR rates in our study were lower than those reported by Ielasi and colleagues and Costopoulos and colleagues (approximately 7.3%). It should be noted that the previous studies were conducted before 2017, while the population in our study were enrolled from 2017 to 2021. We hypothesized that intensive use of antiplatelet and lipid‐lowering drugs contribute to lower TVR rates. Although our study expanded the study population and showed acceptable clinical outcomes, it still needs to be evaluated in prospective studies, such as the ongoing HYPER pilot study, a prospective, nonrandomized, multicenter study aimed at assessing the feasibility, safety, and efficacy of the hybrid DES/DCB approach for the treatment of de novo diffuse disease.

A growing body of evidence supports the value of QFR in assessing the functional relevance of coronary lesions, and has demonstrated good agreement and diagnostic accuracy with fractional flow reserve, which is superior to 2D QCA.^17^ In our study, the post‐PCI QFR value showed the effectiveness of the hybrid strategy. The percentage of patients with post‐PCI QFR value >0.8 in the treated vessels exceeded 95%. Notably, previous studies have not explored the functional impacts of hybrid strategy on prognosis. Recently, Biscaglia et al.^18^ and Kogame et al.^19^ showed that PCI with lower QFR values was associated with an increased vessel‐oriented composite endpoint, with a cut‐off value of 0.9, thereby showing the implication of post‐PCI QFR. In our study, the cut‐off value of 0.9 was also helpful in the discrimination of future events. The estimated rate of MACE excluding periprocedural MI was significantly higher in patients with a post‐PCI QFR < 0.9. This finding provides additional support for the feasibility and efficacy of the hybrid approach from the perspective of functional revascularization, although, the evidence is preliminary, generated by a small number of patients with a limited number of adverse events and should be confirmed in larger studies. We also found that post‐PCI %DS in the DCB‐treated segment was a significant predictor of lower post‐PCI QFR values. This is in line with previous studies indicating that angiographic stenosis parameters are related to QFR values.^3^, ^20^ It also highlights the importance of mitigating post‐PCI %DS in DCB‐treated segments to achieve higher QFR values and better outcomes.

There are several limitations in this study. The first limitation is based on the study design; this was a retrospective case series study without a control group. The sample size was relatively small. Moreover, there was a lack of angiographic follow‐up, which did not allow assessment of the angiographic efficacy of the hybrid strategy. There was also lack of a direct comparison versus conventional strategies. In addition, the relatively limited follow‐up period and selection bias, due to the fact that the treatment strategy for diffuse disease was left to the operator's discretion, prevented us from reaching definitive conclusions regarding clinical outcomes. The second limitation was related to the prognostic value of QFR. The TVR rates were low in the cohort, therefore, the sample was not powered to test the predictive value of QFR for clinical outcomes. There was also a lack of pre‐PCI values that did not permit replication of the findings regarding the prognostic role of pre‐PCI versus post‐PCI values.^21^ Third, the use of intracoronary imaging devices was left to the operator's discretion and was performed in a few cases; future studies are needed to determine whether routine use of intracoronary imaging or in combination with functional evaluation would improve the outcomes of hybrid treatment. Finally, the current study was conducted at a single center and was based on Asian population. The generalizability of our findings to other ethnicities remains to be investigated.

In conclusion, our preliminary results showed that a hybrid strategy using DES and DCB yielded acceptable functional results and was a promising option for the treatment of de novo diffuse CAD. A higher post‐PCI QFR value was associated with better prognosis and might be helpful in guiding this approach.

## CONFLICT OF INTEREST STATEMENT

The authors declare no conflict of interest.

## Supporting information

Supporting information.

## Data Availability

The data that support the findings of this study are available from the corresponding author upon reasonable request.
