# Supplementary material for: Drug‐eluting stent and drug‐coated balloon for the treatment of de novo diffuse coronary artery disease lesions: A retrospective case series study
Source: Clin Cardiol. 2023 Sep 4;46(12):1511–8. doi: 10.1002/clc.24140 (PMC10716315; doi:10.1002/clc.24140)
Supplement: Supplementary file 1 — Supporting information. [file CLC-46-1511-s001.docx]

**Supplemental materials**

**INDEX**

- **Page 2: Supplemental Figure 1. Case of hybrid PCI approach for de novo long diffuse coronary lesions with DCB at proximal segment and DES at distal segment**
- **Page 3: Supplemental Figure 2. Number of vessels in the different post-percutaneous coronary intervention quantitative flow ratio strata**
- **Page 4: Supplemental Figure 3. Case of hybrid PCI approach for de novo long diffuse coronary lesions with suboptimal post-PCI QFR**
- **Page 5: Supplemental Table 1. Baseline characteristics of patients stratified according the post-PCI QFR cut-off (< 0.9)**
- **Page 6: Supplemental Table 2. Lesion and procedural characteristics of vessels stratified according the post-PCI QFR cut-off (< 0.9)**
- **Page 8: Supplemental Table 3. QCA measurements at baseline and after the procedure in vessels stratified according the post-PCI QFR cut-off (< 0.9)**
- **Page 9: Supplemental Table 4. Correlation analysis between post-PCI QFR value and QCA measurements**
- **Page 10: Supplemental Table 5. Logistic regression between QCA measurements and lower post-PCI QFR value (< 0.9)**

**Supplemental Figure 1. Case of hybrid PCI approach for de novo long diffuse coronary lesions with DCB at proximal segment and DES at distal segment**


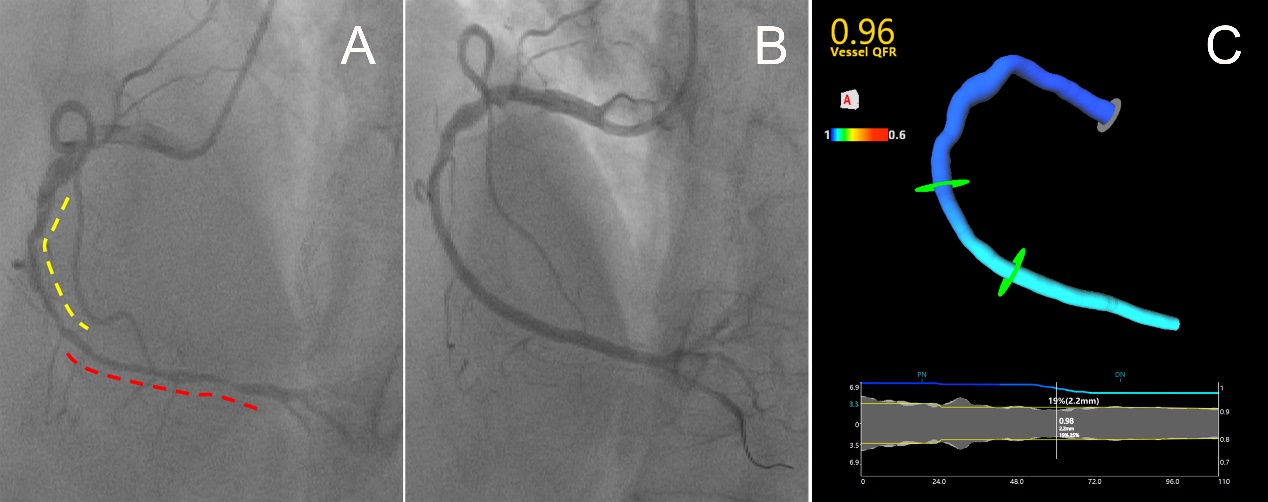


(A) Angiogram in a patient with a de novo long diffuse lesions involving mid-distal right coronary artery. DES- and DCB-treated segments were shown in red and yellow dotted line respectively. (B) Final angiographic result. (C) A three-dimensional model of the coronary was reconstructed based on post-PCI angiogram and result showed an optimal post-PCI QFR value of 0.96.

PCI: percutaneous coronary intervention; DES: drug-eluting stent; DCB: drug-coated balloon; QFR: quantitative flow ratio.

**Supplemental Figure 2. Number of vessels in the different post-percutaneous coronary intervention quantitative flow ratio strata**


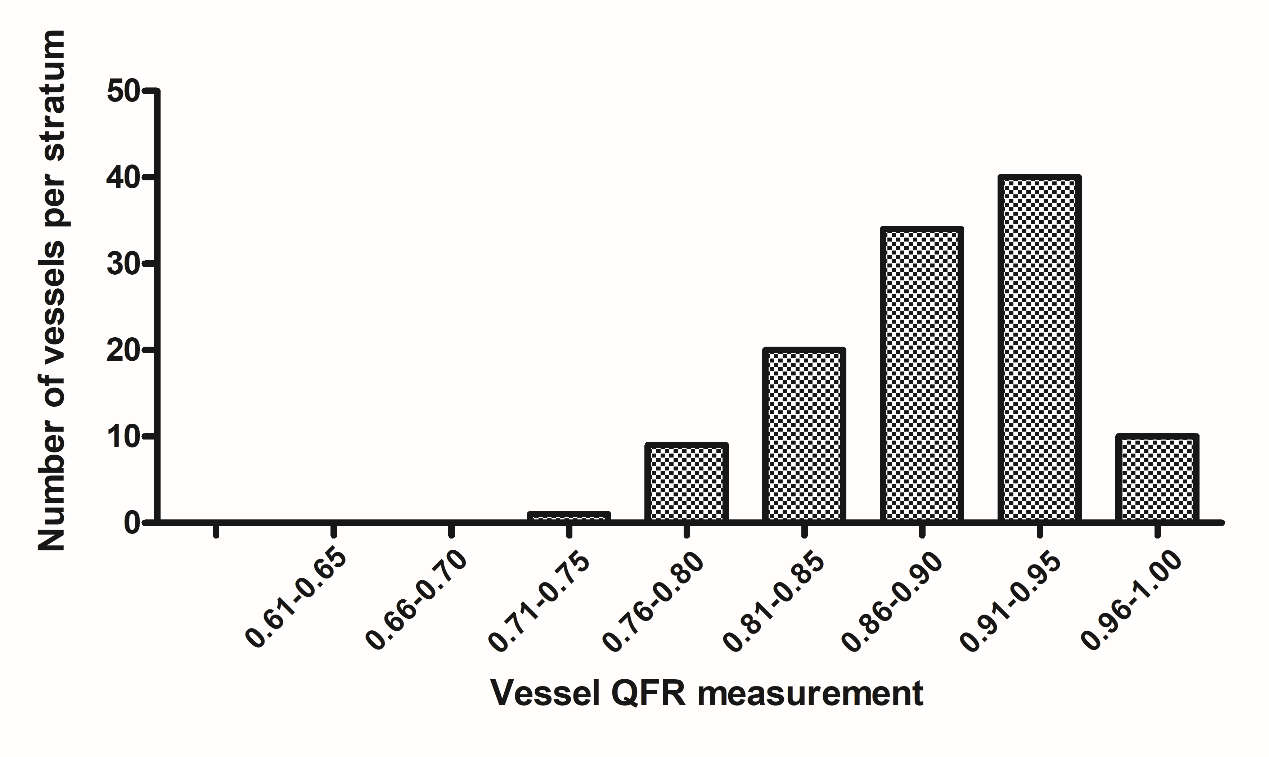


**Supplemental Figure 3. Case of hybrid PCI approach for de novo long diffuse coronary lesions with suboptimal post-PCI QFR**


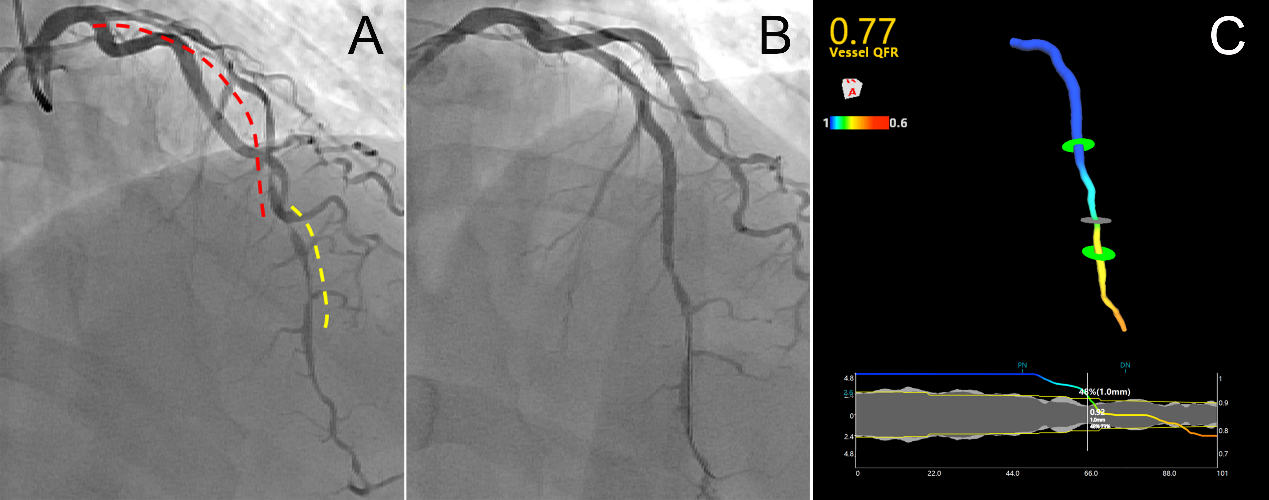


(A) Angiogram in a patient with a de novo long diffuse lesions involving proximal-distal left anterior descending. DES- and DCB-treated segments were shown in red and yellow dotted line respectively. (B) Final angiographic result. (C) A three-dimensional model of the coronary was reconstructed based on post-PCI angiogram and result showed a suboptimal post-PCI QFR value of 0.77. The analysis showed a markedly decrease in QFR value at the DCB-treated segment because of post-procedural diameter stenosis.

PCI: percutaneous coronary intervention; DES: drug-eluting stent; DCB: drug-coated balloon; QFR: quantitative flow ratio.

| **Supplemental Table 1. Baseline characteristics of patients stratified according the post-PCI QFR cut-off (< 0.9)** | | | |
| --- | --- | --- | --- |
| Items | QFR value ≥ 0.9  (n = 73) | QFR value < 0.9  (n =36) | p value |
| Age (y) | 60.5±10.2 | 58.6±10.2 | 0.375 |
| Male | 52 (71.2) | 26 (72.2) | 0.914 |
| BMI (kg/m^2^) | 25.8±2.7 | 26.0±3.1 | 0.786 |
| Hypertension | 52 (71.2) | 26 (72.2) | 0.914 |
| Hyperlipidemia | 63 (86.3) | 29 (80.6) | 0.437 |
| Diabetes | 39 (53.4) | 19 (52.8) | 0.949 |
| Current smoker | 30 (41.1) | 19 (52.8) | 0.249 |
| Family history of CAD | 18 (24.7) | 8 (22.2) | 0.779 |
| Prior MI | 15 (20.5) | 9 (25.0) | 0.598 |
| Prior Stroke | 10 (13.7) | 3 (8.3) | 0.416 |
| Prior PCI | 18 (24.7) | 6 (16.7) | 0.344 |
| Prior CABG | 1 (1.4) | 2 (5.6) | 0.209 |
| Acute coronary syndrome | 23 (31.5) | 17 (47.2) | 0.109 |
| Multivessel CAD | 71 (97.3) | 32 (88.9) | 0.071 |
| SYNTAX score | 21.9±8.9 | 21.4±9.7 | 0.809 |
| LVEF (%) | 60.2±8.1 | 60.1±7.9 | 0.927 |
| NT-pro BNP (pg/mL) | 333.5±494.6 | 280.5±519.2 | 0.606 |
| LDL-C (mmol/L) | 2.2±0.8 | 2.2±0.8 | 0.955 |

Values are expressed as mean ± SD or n (%).

QFR: quantitative flow ratio; PCI: percutaneous coronary intervention; BMI: body mass index; CAD: coronary artery disease; MI: myocardial infarction; CABG: coronary artery bypass graft; SYNTAX: Synergy Between Percutaneous Coronary Intervention with Taxus and Cardiac Surgery; LVEF: left ventricular ejection fraction; NT-pro BNP: N-terminal pro-B-type natriuretic peptide; LDL-C: low-density lipoprotein cholesterol.

| **Supplemental Table 2. Lesion and procedural characteristics of vessels stratified according the post-PCI QFR cut-off (< 0.9)** | | | |
| --- | --- | --- | --- |
|  | QFR value ≥ 0.9  (n = 77) | QFR value < 0.9  (n = 37) | p value |
| Target vessel |  |  | 0.656 |
| Left anterior descending | 30 (39.0) | 17 (45.9) |  |
| Left circumflex | 26 (33.8) | 10 (27.0) |  |
| Right coronary artery | 21 (27.3) | 10 (27.0) |  |
| Location of lesion in treated vessel |  |  | 0.969 |
| Proximal involved | 33 (42.9) | 16 (43.2) |  |
| Mid/distal | 44 (57.1) | 21 (56.8) |  |
| Balloon pre-dilation | 77 (100.0) | 37 (100.0) | - |
| Intracoronary imaging |  |  |  |
| IVUS/OCT | 10 (13.0) | 6 (16.2) | 0.642 |
| Hybrid strategy |  |  | 0.278 |
| DES proximal with DCB distal | 60 (77.9) | 32 (86.5) |  |
| DCB proximal with DES distal | 17 (22.1) | 5 (13.5) |  |
| Device characteristics |  |  |  |
| Number of DES per lesion | 1.5±0.6 | 1.5±0.7 | 0.808 |
| Number of DCB per lesion | 1.1±0.3 | 1.1±0.3 | 0.954 |
| DES length per lesion (mm) | 37.4±17.8 | 34.4±22.3 | 0.451 |
| DCB length per lesion | 23.4±9.4 | 29.1±11.2 | 0.005 |
| DES diameter per lesion | 2.7±0.5 | 2.7±0.3 | 0.941 |
| DCB diameter per lesion | 2.4±0.4 | 2.3±0.4 | 0.100 |
| TIMI flow grade |  |  |  |
| Pre-procedural TIMI flow grade 3 | 50 (64.9) | 24 (64.9) | - |
| Post-procedural TIMI flow grade 3 | 77 (100.0) | 37 (100.0) | - |
| Dissection post-procedural | 19 (24.7) | 12 (32.4) | 0.383 |

Values are expressed as mean ± SD or n (%).

QFR: quantitative flow ratio; PCI: percutaneous coronary intervention; IVUS: intravascular ultrasound; OCT: optical coherence tomography; DES: drug-eluting stent; DCB: drug-coated balloon; TIMI: thrombolysis in myocardial infarction.

| **Supplemental Table 3. QCA measurements at baseline and after the procedure in vessels stratified according the post-PCI QFR cut-off (< 0.9)** | | | |
| --- | --- | --- | --- |
| Items | QFR value ≥ 0.9  (n = 77) | QFR value < 0.9  (n = 37) | p value |
| **DES-treated segment** | | | |
| Pre-procedural QCA |  |  |  |
| Reference vessel diameter (mm) | 2.7±0.3 | 2.7±0.3 | 0.794 |
| Minimal lumen diameter (mm) | 0.4±0.2 | 0.4±0.2 | 0.508 |
| Diameter stenosis (%) | 83.4±7.4 | 84.1±7.5 | 0.622 |
| Lesion length (mm) | 34.0±15.1 | 30.8±18.7 | 0.332 |
| Post-procedural QCA |  |  |  |
| Minimal lumen diameter (mm) | 2.4±0.2 | 2.4±0.2 | 0.630 |
| Diameter stenosis (%) | 10.9±3.1 | 10.5±2.6 | 0.579 |
| Acute gain (mm) | 1.9±0.3 | 1.9±0.3 | 0.523 |
| **DCB-treated segment** | | | |
| Pre-procedural QCA |  |  |  |
| Reference vessel diameter (mm) | 2.4±0.3 | 2.2±0.3 | 0.028 |
| Minimal lumen diameter (mm) | 0.5±0.2 | 0.4±0.1 | 0.030 |
| Diameter stenosis (%) | 80.8±7.0 | 82.9±6.1 | 0.112 |
| Lesion length (mm) | 22.0±8.0 | 26.0±10.1 | 0.024 |
| Post-procedural QCA |  |  |  |
| Minimal lumen diameter (mm) | 2.0±0.3 | 1.7±0.3 | <0.001 |
| Diameter stenosis (%) | 16.5±6.2 | 22.5±7.8 | <0.001 |
| Acute gain (mm) | 1.5±0.2 | 1.3±0.3 | 0.003 |

Values are expressed as means ± SD.

QCA: quantitative coronary angiography; QFR: quantitative flow ratio; PCI: percutaneous coronary intervention; DES: drug-eluting stents; DCB: drug-coated balloon.

| **Supplemental Table 4. Correlation analysis between post-PCI QFR value and QCA measurements** | | |
| --- | --- | --- |
| Variables | r | p value |
| **DES-treated segment** | | |
| **Pre-procedural QCA** | | |
| Reference vessel diameter (mm) | 0.056 | 0.557 |
| Minimal lumen diameter (mm) | 0.072 | 0.447 |
| Diameter stenosis (%) | 0.040 | 0.674 |
| Lesion length (mm) | 0.151 | 0.108 |
| **Post-procedural QCA** | | |
| Minimal lumen diameter (mm) | 0.074 | 0.436 |
| Diameter stenosis (%) | 0.071 | 0.453 |
| Acute gain (mm) | 0.068 | 0.475 |
| **DCB-treated segment** | | |
| **Pre-procedural QCA** | | |
| Reference vessel diameter (mm) | 0.161 | 0.087 |
| Minimal lumen diameter (mm) | 0.112 | 0.233 |
| Diameter stenosis (%) | 0.057 | 0.546 |
| Lesion length (mm) | 0.194 | 0.039 |
| **Post-procedural QCA** | | |
| Minimal lumen diameter (mm) | 0.349 | <0.001 |
| Diameter stenosis (%) | 0.422 | <0.001 |
| Acute gain (mm) | 0.317 | 0.001 |

QCA: quantitative coronary angiography; QFR: quantitative flow ratio; PCI: percutaneous coronary intervention; DES: drug-eluting stents; DCB: drug-coated balloon.

| Supplemental Table 5. Logistic regression between QCA measurements and lower post-PCI QFR value (< 0.9) | | | | |
| --- | --- | --- | --- | --- |
|  | **Univariate** | | **Multivariate** | |
| Variables | OR (95%CI) | p value | OR (95%CI) | p value |
| DES-treated segment | | | | |
| Pre-procedural QCA | | | | |
| Reference vessel diameter (mm) | 0.83 (0.21-3.32) | 0.792 | 1.29 (0.19-8.88) | 0.798 |
| Diameter stenosis (%) | 1.01 (0.96-1.07) | 0.618 | 1.02 (0.95-1.11) | 0.563 |
| Lesion length (mm) | 0.99 (0.96-1.01) | 0.330 | 0.97 (0.94-1.01) | 0.121 |
| Post-procedural QCA | | | | |
| Diameter stenosis (%) | 0.96 (0.84-1.11) | 0.576 | 0.95 (0.82-1.11) | 0.538 |
| DCB-treated segment | | | | |
| Pre-procedural QCA | | | | |
| Reference vessel diameter (mm) | 0.19 (0.04-0.86) | 0.031 | 0.15 (0.02-1.01) | 0.051 |
| Diameter stenosis (%) | 1.05 (0.99-1.13) | 0.116 | 0.99 (0.90-1.09) | 0.877 |
| Lesion length (mm) | 1.05 (1.01-1.10) | 0.029 | 1.04 (0.98-1.10) | 0.160 |
| Post-procedural QCA | | | | |
| Diameter stenosis (%) | 1.13 (1.06-1.20) | < 0.001 | 1.13 (1.06-1.21) | < 0.001 |

Data are presented as OR (95% CI).

QCA: quantitative coronary angiography; QFR: quantitative flow ratio; PCI: percutaneous coronary intervention; OR: odds ratio; CI: confidence interval; DES: drug-eluting stents; DCB: drug-coated balloon.
